# Inhaled Drug Therapy-Associated Adverse Reactions in Obstructive Respiratory Diseases: A Review of a Decade of Reporting to the Portuguese Pharmacovigilance System

**DOI:** 10.3390/ijerph182312411

**Published:** 2021-11-25

**Authors:** Willy Fonseca, Cristina Monteiro, Luís Taborda-Barata

**Affiliations:** 1 Faculty of Health Sciences, University of Beira Interior, Avenida Infante D. Henrique, 6200-506 Covilhã, Portugal; a32953@fcsaude.ubi.pt (W.F.); tabordabarata@fcsaude.ubi.pt (L.T.-B.); 2 UFBI—Pharmacovigilance Unit of Beira Interior, University of Beira Interior, Avenida Infante D. Henrique, 6200-506 Covilhã, Portugal; 3 Department of Immunoallergology, Cova da Beira University Hospital Centre, Quinta do Alvito, 6200-001 Covilhã, Portugal

**Keywords:** adverse drug reactions, asthma, COPD, pharmacovigilance, inhalation devices, safety

## Abstract

Inhaled medication used for treatment of chronic obstructive lung diseases (asthma, chronic obstructive pulmonary disease-COPD, and Asthma-COPD overlap) may be associated with adverse drug reactions (ADRs). The aim of this study was to characterise spontaneous reports (SRs) of suspected ADRs received by the Portuguese Pharmacovigilance System (PPS), from 2007 to 2017. Methods: Retrospective observational study of SRs associated with single substance and combination inhalers, analysed in terms of pharmacological class of the involved drugs, sex and age range of the involved patients, and seriousness and type of ADRs. Results: 230 SRs were analysed, accounting for a total of 599 suspected ADRs. Inhaled corticosteroid/long-acting beta-2 agonist combination had the highest frequency in SRs (32.2%) and in ADRs (32.7%). There was a slight predominance in men (51.3%) and non-elderly adults were the most affected age group (39.1%). Most SRs were serious (70.4%). In total, “respiratory, thoracic and mediastinal diseases” ADRs were the most reported (19.5%), with “dyspnea” being the most frequent (4.8%). Conclusions: Most SRs were associated with controller medications and were expected. Most ADRs involved non-elderly adults, were serious and of respiratory nature and many were due to overuse of reliever medication.

## 1. Introduction

Asthma and chronic obstructive pulmonary disease (COPD) belong to the group of obstructive lung diseases (OLDs). These are characterised by an increase in resistance to airflow due to partial or complete obstruction in the airways, as well as by a variable inflammatory component [1,2,3]. More recently, the asthma-COPD overlap (ACO) entity has been described, although it does not yet have a universally accepted definition. ACO more commonly involves patients who are usually older than 40 years and who have a persistent airflow obstruction with both asthma and COPD features [2,4]. ACO is, thus, also part of OLDs. The main treatments for OLDs involve medication delivered via inhaler devices. Inhaled medications used belong to the following classes: inhaled corticosteroids (ICS), β2-adrenergic receptor agonists (with short-acting β2 agonists (SABA) and long-acting β2 agonists (LABA) subclasses) and muscarinic receptor antagonists (with short-acting muscarinic antagonist (SAMA) and long-acting muscarinic antagonist (LAMA) subclasses). ICS and long-acting bronchodilators (LABA and LAMA) are used as controllers while short-acting bronchodilators (SABA and SAMA) are used as relievers [2,3,4].

As with any drug, inhaled therapy for OLDs is associated with adverse drug reactions (ADRs), especially at high doses (high dose in limits of recommend dose). An ADR is defined as a noxious and unintended response to a medicine, whether the product is being used within or outside the terms of the marketing authorisation (off-label use, overdose, misuse, abuse and medication errors) [5]. There must, of course, be a suspected causality between the reaction and the drug [5]. Known reactions associated with inhalers have been reported in pre-marketing clinical trials and case reports [6,7,8]. ADRs associated with ICS may be local, by deposition in the oropharynx, or systemic when absorbed into the systemic circulation through the lungs or gastrointestinal tract, when swallowed [6]. The most frequently reported ICS-associated local ADRs are oropharyngeal candidiasis, dysphonia and cough during inhalation [6]. Systemic ADRs occur primarily with high doses of ICS, and include easy bruising, adrenal suppression (very rare), thinning of the skin, increased risk of cataracts and glaucoma (very rare), decreased bone density (very rare), psychiatric effects (rare) and, in COPD, increased risk of pneumonia and tuberculosis [6]. However, the patients have negligible systemic side effects at the doses most patients require, for the control of the disease [6]. β2-agonists also have systemic absorption through the lungs or gastrointestinal tract if they are swallowed. Most of the described β2-agonist-associated ADRs are sympathomimetic, with the most common ones being increased heart rate, palpitations, transient decrease in oxygen partial pressure in arterial blood, hyperglycaemia, hypokalaemia, cardiac arrhythmias and tremors [7]. Muscarinic antagonists are poorly absorbed, and are therefore associated with few systemic effects. The most common ADRs are dry mouth, constipation, blurring of vision, urinary difficulty/retention and cardiac effects (increased heart rate, arrhythmias and angina) [8].

There are few studies focused on spontaneous ADR reports to Pharmacovigilance Systems [9,10], and there is no study published in Portugal, to the best of our knowledge, relating the inhaled therapy and ADRs in COPD with data of Pharmacovigilance system. Pharmacovigilance is responsible for detection, assessment, and prevention of ADR, in post-marketing authorisation phase [11]. Spontaneous reporting is a source of information on previously unknown, rare occurring and serious reactions that are not detected in controlled studies [12]. Taking into account that the use of inhaled therapy for OLDs is widespread, the importance of spontaneous ADRs reports in Pharmacovigilance and the absence of studies in Portugal, the aim of this study was to analyse the suspected ADRs spontaneously reported to the Portuguese Pharmacovigilance System (PPS) associated with inhaled therapy used in obstructive lung diseases from 2007 to 2017.

## 2. Materials and Methods

### 2.1. Study Type, Setting and Data Source

A retrospective and observational research study based on the collection and analysis of spontaneous reports (SRs) of suspected ADRs associated with inhaled medications used in obstructive lung diseases (COPD, Asthma and ACO), received by the PPS from 2007 to 2017, was performed. The PPS is coordinated by the National Authority of Medicines and Health Products, I.P. (INFARMED). As data are anonymous, Ethics Committee approval was not needed.

We analysed SRs indirectly received from marketing authorization holders, SRs directly received from healthcare professionals (physicians, pharmacists, nurses and other healthcare professionals) and SRs directly received from non-healthcare professionals (consumers or other non-healthcare professionals).

### 2.2. Inhaler-Delivered Drugs Selected for the Study

Based on the WHO Anatomical Therapeutic Chemical (ATC) Classification System [13], the drugs belonging to the following subgroups were selected: R03AC (Selective β2-adrenoreceptor agonists), R03AK (Adrenergics in combination with corticosteroids or other drugs, excl. anticholinergics), R03AL (Adrenergics in combinations with anticholinergics incl. triple combinations with corticosteroids), R03BA (Glucocorticoids) and R03BB (Anticholinergics). The substances are identified by their International Nonproprietary Name (INN).

We analysed the suspected ADR associated with 20 substances or their combinations of three pharmacological classes: muscarinic antagonists (MA) (with the long-acting muscarinic antagonists (LAMA) and short-acting muscarinic antagonists (SAMA) subclasses), β2 adrenergic receptor agonists/β2-agonists (β2A) (with the short-acting β2 agonists (SABA) and long-acting β2 agonists (LABA) subclasses) and inhaled corticosteroids (ICS). Both single-substance inhalers (inhalers with only one active substance) and combination inhalers (inhalers with more than one active substance) are present. Table 1 shows how the inhalers were grouped. The term “multiple inhalers” refers to cases where there was more than one suspected inhaler.

Duplicate SRs and those whose routes of administration of the drug are different from those intended in this study were excluded. 

### 2.3. Parameters Used for Analysis

Based on the information found in the SRs, an analysis focused on the following parameters was performed: patient’s sex and age group, pharmacological classes of suspected medicines and type and seriousness of suspected ADRs. SRs with the following criteria were classified as serious: fatal, life-threatening, requiring hospitalization or prolongation of existing hospitalization, resulting in persistent or significant disability/incapacity, resulting in a congenital anomaly/birth defect or considered clinically important [14]. All other SRs were classified as non-serious.

The frequency distribution of SRs over the involved time period was also represented, adjusted to the estimated population with asthma and COPD. It was calculated by summing up the estimated population with asthma, considering the estimated prevalence of 6.8% [15], with the estimated population with COPD, considering the estimated prevalence of 5.34% [16]. The general data for the Portuguese population for each year were obtained from the National Institute of Statistics (INE) website [17].

Each SR corresponds to a patient. However, there may be more than one ADR in each SR. They were classified according to sex and divided into three age groups: children (0–17 years), adults (18–64 years) and the elderly (65 years and older).

Each suspected ADR was analysed in accordance with the Preferred Term (PT) and System Organ Class (SOC) of the Medical Dictionary for Regulatory Activities (MedDRA) terminology from which it was coded [18].

### 2.4. Statistical Analysis

Data analysis was performed using descriptive statistics. Variables under study are qualitative and are described in absolute (*n*) and relative (%) frequencies. Chi-Square Test (*χ*^2^) was performed to examine the relation between seriousness and sex, and between seriousness and age group of the patients. *p*-value (*p*) equal or less than 0.05 was regarded statistically significant.

Data processing was made using the software programs Microsoft^®^ Office^®^ Excel^®^ 365 (Microsoft Corporation, Redmond, WA, USA) and IBM^®^ SPSS^®^ (Statistical Package for the Social Sciences) version 22.0 (IBM Corporation, Armonk, NY, USA).

## 3. Results

### 3.1. Selection of Reports and Reporting Time Trends

We started with a total of 271 SR. After all duplicate reports were eliminated, we obtained 254 SR. Finally, after excluding SRs of suspected drugs with a different administration route from those intended in this study, we were left with 230 SR. We considered SRs that had no information on the therapeutic indication and also those with different therapeutic indications (e.g., wheezing). A total of 599 suspected ADRs were identified in the 230 SR.

In general, there was an increase in reporting over the years, with more than half (58.7%) occurring in the last 4 years (2014–2017). The mean was 2 SRs per 1000 inhabitants with an OLD per year (Table 2).

### 3.2. Pharmacological Classes Associated with Adverse Drug Reactions

In terms of pharmacological classes, the ICS/LABA combination showed the highest frequency in both SR (*n* = 74, 32.17%) and in suspected ADRs (*n* = 196, 32.72%). The budesonide/formoterol combination obtained the highest frequency of SRs (*n* = 34; 14.78%) while the fluticasone/salmeterol combination had the highest record in terms of suspected ADRs (*n* = 91, 15.19%), both belonging to the ICS/LABA class (Table 3).

### 3.3. Demographics Features of Most Frequent Spontaneous Reports

Non-elderly adults were the most affected (SRs *n* = 90, 39.1%). Reports on LAMA, SAMA and LAMA/LABA were more frequent in elderly adults. Reports on LABA, ICS, SAMA/SABA and ICS/LABA were more frequent in non-elderly adults. Finally, reports on SABA were more frequent in children. Information on the patient’s age group was unknown in 37 SRs (16.1%). There was a slight male predominance (reports *n* = 118, 51.3%). Reports related to males were more frequent in all classes, except for SABA, SAMA/SABA and ICS/SABA. Information on the gender of patients was unknown in five cases (2.2%) (Table 4).

### 3.4. Source of Spontaneous Reports

Among the 230 SRs, 143 (62.2%) were indirectly received from marketing authorization holders, with the majority being serious (119); 36 (15.7%) were directly received by pharmacists, followed by physicians (*n* = 26, 11.3%), consumers or other non-healthcare professionals (*n* = 22, 9.6%), other healthcare professionals (*n* = 2, 0.9%) and nurses (*n* = 1, 0.4%). Indirectly received SRs were the most frequent in all years under study. In directly received SRs, pharmacist reported the most SRs over the years, except for 2010, 2012 and 2017 where physicians were the health professionals who submitted the highest number of notifications. Consumers notified more in 2015 and 2016. Physicians were the health professionals that notified more serious SRs in the considered direct reports.

### 3.5. Characteristics Associated with Serious Sponateneous Reports

Most SRs were labelled as serious (*n* = 162, 70.4%). SRs labelled as serious were more frequent in all classes, except for the SAMA/SABA combination (Table 5).

Of the 162 serious SRs, 117 had the criteria of clinically important, 19 hospitalisation, 9 disability, 5 clinically important and hospitalisation, 3 life-threatening, 2 clinically important and life-threatening, 2 death, 1 life-threatening and disability, 1 hospitalisation and life-threatening, 1 clinically important and disability, 1 hospitalisation and death and 1 congenital anomaly. SR with the criteria hospitalisation and the criteria disability were both more frequent in LABAs. There were three fatal cases, one being initially related to an ICS (budesonide), presumably being due to acute kidney injury and overdose, and the other two to a LAMA (glycopyrronium bromide), presumably being due to lung cancer and bronchopulmonary aspergillosis, respectively. However, the experts concluded afterwards that none had causality with these drugs. 

SRs labelled as serious were the most frequent ones in both sexes and all age groups. Males and non-elderly adults had the highest absolute frequency. The relation between seriousness and age group was significant, *χ*^2^(2) = 8.06, *p* = 0.018, which means that SRs labelled as serious were more likely to be associated with non-elderly adults. The relation between seriousness and sex group was not significant, *χ*^2^(1) = 1.12, *p* = 0.289.

### 3.6. Most Frequently Reported Adverse Drugs Reactionss According to System Organ Class

The suspected ADRs included in the “Respiratory, thoracic and mediastinal disorders” SOC were the most frequently reported ones (*n* = 117, 19.5%) (Table 6). “Dyspnoea” was the most frequent suspected ADR reported (*n* = 29; 4.8%) in the total of the analysed SRs. According to the SOC, by pharmacological class “Respiratory, thoracic and mediastinal disorders” were the most frequently reported ADRs with SAMA, LABA and ICS/LABA inhalers, whereas “General disorders and administration site conditions” were the most commonly reported ADRs with ICS and LAMA/LABA inhalers (Table 6).

In terms of suspected serious ADRs, “chest pain” and “headache” were the most frequently reported ones with LAMA, and “dyspnoea” was the most frequently reported serious ADR with SAMA, LABA, LAMA/LABA and ICS/LABA. It should be noted that “overdose” was the most frequent suspected serious ADRs with SABA, whereas with ICS, it was “Cushing’s syndrome”.

In terms of serious suspected ADRs, “dyspnoea” was the most frequently reported in both sexes and in all age groups, except in children where “peripheral oedema” was the most frequent one.

## 4. Discussion

In this first Portuguese study of reporting of ADRs to the Portuguese Pharmacovigilance System over a 10-year-long period, regarding inhaler-delivered medication for obstructive lung diseases, we were able to determine the overall reporting frequency, the demographic features of reported cases and the most frequently reported ADRs in terms of severity, clinical features and specific frequency, associated with each inhaler medication type. This is one of few international pharmacovigilance reports of this nature.

ADRs are a major public health problem worldwide, being a considerable cause of mortality, morbidity and financial cost [19]. They may occur with any medicinal product, and therefore, inhaled therapy used in OLDs is no exception. Spontaneous ADRs reporting is the cornerstone of post-marketing drug safety surveillance which is an important part of Pharmacovigilance. Due to the fact that such monitoring occurs in real world conditions, it becomes one of the best methods to generate signals on rare and new ADRs, which were not detected in pre-marketing controlled clinical trials [12]. In our study, 230 SRs referring to 599 suspected ADRs were analysed. This value constitutes about 0.6% of the total SRs received by the PPS from 2007 to 2017 [20]. The inclusion of SRs in which the therapeutic indication was not identified or had a different indication contributed to the identification of rare reactions.

In the period of time analysed in our study, there was an overall trend towards increasing reporting. This may be related to the overall increase in the total of spontaneous ADRs reports received by PPS in recent years [20]. The true incidence rate of ADRs cannot be correctly determined because of the lack of information on the actual number of patients exposed to the drugs being studied. In this study, a speculation of this rate was made by adjusting the number of annual SRs to an estimate of the Portuguese population with an OLD based on the prevalence values of asthma and COPD, obtaining a mean of about 2 SRs per 1000 per year.

In general, controllers (ICS, LABA, LAMA, ICS/LABA combination and LABA/LAMA combination) had a higher frequency of SRs compared to relievers (SABA, SAMA and SABA/SAMA combination) (81.7% vs. 13%). According to guideline recommendations [2,3], controllers are used daily as maintenance therapy while relievers must be used only as needed for a quick symptom relief. Because they are more regularly used, one may expect a higher frequency of ADRs with controllers.

Although, in general, women and older patients are regarded as the most susceptible groups to ADRs [21], in our study the suspected ADRs were more frequently reported for males and non-elderly adults. In specific terms of inhaled therapy for OLDs, cross-sectional observational studies have shown that women may [22] or may not [23] be more prone than men to developing side effects from inhaled medication, namely corticosteroids. Several reasons may account for these discrepancies. Firstly, it should be noted that in some reports in our study there was a lack of information about the patient’s sex (2.2%) and age group (16.1%), and this may have biased our results. Secondly, pharmacovigilance studies depend upon various features of the reporting population, namely awareness of reporting, readiness to report, or other factors. These aspects may lead to varying results in terms of gender, across studies. Thirdly, asthma and COPD may have differences in prevalence in terms of sex. In asthma there seems to be a male predominance up to 18 years of age and female predominance in adulthood [24], whereas COPD apparently has a male prevalence in all age groups [25]. Since most patients in our study were 18 years old or older, it is tempting to assume that ADRs in people with asthma were more frequent in women and in COPD were more frequent in men, and an attempt to explain the greater overall prevalence of ADRs in men could be tried by analysing the therapeutic indications of the drugs to see if there was a greater proportion of COPD. However, because we included SRs in which the therapeutic indication was not identified or was different from those intended in this study, it is difficult to make this assumption.

In terms of age, although asthma is more prevalent in children [26] and COPD is more prevalent in older patients [16], in this study ADRs were more common in non-elderly adults, and this can be partly explained by the fact that this is the age group that makes up the majority of Portuguese population [17].

Suspected serious ADRs were the most reported ones (70.4%). This may in part be related to the fact that serious reactions are more likely to be reported, as was observed in a previous pharmacovigilance study involving general reporting of ADRs with any medication, in Portugal [27]. This observation can be found in other studies on spontaneous reports of ADR associated with other pharmacological classes [28,29,30]. In this context, it is also important to mention that health professionals seem to be particularly prone to reporting only serious ADRs, which explains the high number of serious cases reported [26,27,28,29,30]. Most cases were reported by marketing authorisation holders, which should record all suspected adverse reactions related to their drugs, and directly by healthcare professionals, particularly pharmacists and physicians, similarly to what occurred in other studies [26,27,28,29,30]. Despite consumers also being able to report ADR, many patients may still be unaware of how to recognise ADRs and how to report them [28].

With the data of this work, we can verify that the probability of a reaction being serious is not influenced by the sex group, being, however, influenced by the age group. In a prospective, observational, one-year-long real-life Bulgarian study of suspected ADRs associated with COPD therapy reported to the Bulgarian Drug Agency (BDA) [9] and in a Danish study of suspected reported ADRs associated with asthma medication licensed for paediatric use located in the European ADR database (EudraVigilance) [10], the majority of ADRs analysed were also serious.

Across medications, the majority of the reported ADRs were of the “respiratory, thoracic and mediastinal disorders” type, followed by “general disorders and administration site conditions” and “skin and subcutaneous tissue disorders”. In the Bulgarian study [9], the most reported suspected ADRs types were “nervous system” followed by “respiratory system” and “cardiovascular system”. However, this study included drugs from other classes, such as roflumilast (a long-acting inhibitor of the enzyme phosphodiesterase-4), aminophylline and theophylline (xanthine derivates). In the Danish study [10], the majority of reported ADRs were of the “psychiatric disorders” type, followed by ‘‘respiratory, thoracic and mediastinal disorders’’ and ‘‘skin and subcutaneous disorders”. Montelukast (a leukotriene receptor antagonist) was also included in this study.

As previously mentioned, the majority of the reported ADRs were also of the “respiratory, thoracic and mediastinal disorders” type of SOC. Although there may be adverse respiratory effects with any of the inhaled medication types, respiratory SOC, particularly in terms of “dyspnoea” ADR may be due to drug ineffectiveness. In fact, in our report, there were many cases of drug ineffectiveness and product quality issues leading to symptoms such as dyspnoea and cough, and even exacerbations. At least in part, such ineffectiveness may be due to errors in inhaler device handling which are associated with reduced drug delivery to the lungs and ineffectiveness of treatment, thereby resulting in suboptimal symptom control in asthma or COPD [31,32,33,34,35]. Further, the overall error rate appears to be high across all inhaler devices, ranging from 50 to 100% [32]. In a study conducted in four community pharmacies in the Portugal central region, in which 67 adult patients with asthma or COPD were invited to demonstrate their technique, 87% of the participants had at least one error [34]. In another study, carried out in the same region, inhaler technique errors were very common in both elderly and non-elderly patients with asthma or COPD [35].

There are multiple factors involved, related to the device (manipulation, dexterity and hand strength required and hand-lung coordination), consumers (physical capabilities, health beliefs/beliefs about medications, adherence and device preference) and healthcare professionals (demonstration of correct inhaler technique and frequent reviews of the patient’s technique) [33].

In our study, most of the frequently reported serious suspected ADRs were expected (84.5%), since they are described in the summary of product characteristics (SmPC) of, at least, one drug of the pharmacological class. Regulatory authorities should be encouraged to evaluate unexpected ADRs and to update SmPCs regularly.

There were three serious cases of drug overdose associated with SABAs, which may indicate that patients did not have an optimal control of their disease, leading to overuse of quick reliever medications. This finding is also described in the aforementioned Danish and Bulgarian studies [9,10]. As described, SABA overuse has been associated with worse disease control and more frequent symptoms and exacerbations in both asthma and COPD [36,37]. In an American study with a sample of 416 asthmatic patients, 27% were SABA overusers [36]. In another American study with a sample of 32 COPD patients, nearly 50% overused their SABA medications at least once during the period of observation [37]. In a Portuguese study aimed at describing SABA overuse using the Portuguese Electronic Medical Prescription (PEM) database, 1.9% of all patients on whom SABA was prescribed were overusers [38]. The causes of overuse are multifactorial and complex, and an important factor is the fact that patients rely on the quick effect of SABA to manage their COPD and persistent asthma instead of the recommended controller medications. As can be expected, overuse and overdosing are associated with an increased risk of ADRs.

To the best of our knowledge, this is the first analysis of suspected ADRs spontaneously reported to a central pharmacovigilance system, focused only on the inhaled therapy of obstructive lung diseases and including all age groups. However, our study has various limitations. The first one involves underreporting bias. Indeed, it is estimated that only 6% of all ADRs are reported [39]; thus, it is impossible to determine the true ADR incidence rate, a value that can only be speculated. Secondly, the variability of the quality of the reported data has to be taken into consideration. In fact, since only four minimum criteria are needed to submit an ADR report as an Individual Case Safety Report (ICSR), a significant proportion of the reports lack critical information. Thirdly, relevant information such as the type of inhalers being used and the dose used could not be significantly retrieved from the reports. Finally, the lack of full records of the presence of eventual confounding factors, such as underlying medical disorders and concomitant medications, hinders the establishment of causality between adverse reaction and suspected drug.

Nevertheless, in spite of the obvious limitations, our study is novel and yields very relevant information obtained from real-life context of inhaler drug usage in obstructive lung diseases.

## 5. Conclusions

The incidence of inhaled therapy spontaneous ADR reports has increased over time. Suspected ADR were more associated with controller medication, and most of the reports and ADRs were related with the LABA/ICS combination class. There was a slight male predominance and non-elderly adults were the age group more affected. The majority of the reports were labelled as serious. Respiratory tract was the most frequently affected organ system, with dyspnoea as the most reported reaction. There were many cases of suspected device error leading to suboptimal therapy effectiveness.

In order to further analyse the context of inhaler-delivered medication-associated ADRs in obstructive lung diseases, further pharmacovigilance studies should be carried out, namely in European-wide terms, and actions focusing on education regarding reporting of ADRs should be implemented not only in health care professionals but also in the general population.

## Figures and Tables

**Table 1:** Single-substance and combination inhalers.

**Single-Substance Inhalers**						
**Muscarinic Antagonists (MA)**			**β2-Agonists (β2A)**			**ICS**
**LAMA**	**SAMA**		**LABA**	**SABA**		
Aclidinium Br.	Ipratropium Br.		Formoterol	Salbutamol		Beclometasone
Glycopyrronium Br.	-		Indacaterol	Terbutaline		Budesonide
Tiotropium Br.	-		Salmeterol	-		Fluticasone
**Combination Inhalers**						
**LAMA/LABA**		**SAMA/SABA**			**ICS/LABA**	
Aclidinium Br./Formoterol		Ipratropium Br./Salbutamol			Budesonide/Formoterol	
Glycopyrronium Br./Indacaterol		-			Fluticasone/Salmeterol	
Tiotropium Br./Olodaterol		-			Fluticasone/Vilanterol	
Umeclidinium Br./Vilanterol		-			-	

Br: Bromide; LAMA: Long Acting Muscarinic Antagonist; SAMA: Short Acting Muscarinic Antagonist; LABA: Long Acting Beta 2 Agonist; SABA: Short Acting Beta 2 Agonist; ICS: Inhaled Corticosteroid.

**Table 2:** Spontaneous Reports (SRs) frequency distribution of SRs over the years, adjusted to the estimated Portuguese population with asthma and asthma, chronic obstructive pulmonary disease.

Year	SRs *n* (%)	SRs/1000 Inhabitants with an OLD
2007	9 (3.9)	0.9
2008	7 (3)	0.7
2009	7 (3)	0.7
2010	15 (6.5)	1.4
2011	19 (8.3)	1.8
2012	24 (10.4)	2.3
2013	14 (6.1)	1.3
2014	28 (12.2)	2.7
2015	45 (19.6)	4.3
2016	25 (10.9)	2.4
2017	37 (16.1)	3.6
**Total**	230	2/Year

OLDs: obstructive lung diseases; SRs: Spontaneous Reports.

**Table 3:** Frequency of Spontaneous Reports (SRs) and suspected Adverse Drug Reactions (ADRs) by pharmacological class.

Pharmacological Classes		Pharmacological Subclasses	SRs *n* (%)	ADRs *n* (%)
**Single-substance Inhalers**	**Muscarinic Antagonists (MA)**	**LAMA** Aclidinium Br. Glycopyrronium Br. Tiotropium Br.	32 (13.9)	76 (12.7)
		**SAMA** Ipratropium Br.	14 (6.1)	44 (7.3)
	**β2-agonists (β2A)**	**LABA** Formoterol Indacaterol Salmeterol	33 (14.3)	78 (13)
		**SABA** Salbutamol Terbutaline	15 (6.5)	32 (5.3)
	-	**ICS** Beclometasone Budesonide Fluticasone	28 (12.2)	84 (14)
**Combination Inhalers**	-	**LAMA/LABA** Aclidinium Br./Formoterol Glycopyrronium Br./Indacaterol Tiotropium Br./Olodaterol Umeclidinium Br./Vilanterol	21 (9.1)	62 (10.4)
		**SAMA/SABA** Ipratropium Br./Salbutamol	1 (0.4)	3 (0.5)
		**ICS/LABA** Budesonide/Formoterol Fluticasone/Salmeterol Fluticasone/Vilanterol	74 (32.2)	196 (32.7)
		Multiple inhalers	12 (5.2)	24 (4)
		**Total**	230 (100)	599 (100)

LAMA: Long Acting Muscarinic Antagonist; SAMA: Short Acting Muscarinic Antagonist; LABA: Long Acting Beta 2 Agonist; SABA: Short Acting Beta 2 Agonist; ICS: Inhaled Corticosteroid.

**Table 4:** Demographic aspects of the Spontaneous Reports by pharmacological classes.

Class	Sex			Age Group (Years)			
	Male *n* (%)	Female *n* (%)	NI *n* (%)	0–17 *n* (%)	18–64 *n* (%)	≥65 *n* (%)	NI *n* (%)
LAMA	21 (65.6)	11 (34.4)	0 (0)	0 (0)	6 (18.8)	23 (71.9)	3 (9.4)
SAMA	11 (78.6)	3 (21.4)	0 (0)	1 (7.1)	4 (28.6)	7 (50)	2 (14.3)
LABA	17 (51.5)	14 (42.4)	2 (6.1)	2 (6.1)	12 (36.4)	11 (33.3)	8 (24.2)
SABA	5 (33.3)	10 (66.7)	0 (0)	8 (53.3)	5 (33.3)	0 (0)	2 (13.3)
ICS	17 (60.7)	11 (39.3)	0 (0)	11 (39.3)	13 (46.4)	4 (14.3)	0 (0)
LAMA/LABA	14 (66.7)	7 (33.3)	0 (0)	0 (0)	3 (14.3)	9 (42.9)	9 (42.9)
SAMA/SABA	0 (0)	1 (100)	0 (0)	0 (0)	1 (100)	0 (0)	0 (0)
ICS/LABA	29 (39.2)	42 (56.8)	3 (4.1)	4 (5.4)	40 (54.1)	19 (25.7)	11 (14.9)
Multiple Inhalers	4 (33.3)	8 (66.7)	0 (0)	0 (0)	6 (50)	4 (33.3)	2 (16.7)
**Total**	118 (51.3)	107 (46.5)	5 (2.2)	26 (11.3)	90 (39.1)	77 (33.5)	37 (16.1)

LAMA: Long Acting Muscarinic Antagonist; SAMA: Short Acting Muscarinic Antagonist; LABA: Long Acting Beta 2 Agonist; SABA: Short Acting Beta 2 Agonist; ICS: Inhaled Corticosteroid; NI: not identified.

**Table 5:** Spontaneous Reports seriousness by pharmacological classes.

Pharmacological Class	Serious *n* (%)	Non-Serious *n* (%)
LAMA	22 (68.8)	10 (31.3)
SAMA	14 (100)	0 (0)
LABA	30 (90.9)	3 (9.1)
SABA	10 (66.7)	5 (33.3)
ICS	22 (78.6)	6 (21.4)
LAMA/LABA	13 (61.9)	8 (38.1)
SAMA/SABA	0 (0)	1 (100)
ICS/LABA	42 (56.8)	32 (43.2)
Multiple inhalers	9 (75)	3 (25)
**Total**	162 (70.4)	68 (29.6)

LAMA: Long Acting Muscarinic Antagonist; SAMA: Short Acting Muscarinic Antagonist; LABA: Long Acting Beta 2 Agonist; SABA: Short Acting Beta 2 Agonist; ICS: Inhaled Corticosteroid.

**Table 6:** Most frequently reported Adverse Drug Reactions (ADRs) according to System Organ Classes (SOC), by pharmacological class.

SOC Where the Suspected ADR Were Included	*n*
**LAMA**	
Nervous system disorders	12
General disorders and administration site conditions	11
**SAMA**	
Respiratory, thoracic and mediastinal disorders	11
Cardiac disorders	7
**LABA**	
Respiratory, thoracic and mediastinal disorders	25
Cardiac disorders	10
**SABA**	
Injury, poisoning and procedural complications	7
General disorders and administration site conditions	6
**ICS**	
General disorders and administration site conditions	16
Skin and subcutaneous tissue disorders	15
**LAMA/LABA**	
General disorders and administration site conditions	12
Respiratory, thoracic and mediastinal disorders	7
**SAMA/SABA**	
Cardiac disorders	1
Nervous system disorders	1
Respiratory, thoracic and mediastinal disorders	1
**ICS/LABA**	
Respiratory, thoracic and mediastinal disorders	48
General disorders and administration site conditions	38

LAMA: Long Acting Muscarinic Antagonist; SAMA: Short Acting Muscarinic Antagonist; LABA: Long Acting Beta 2 Agonist; SABA: Short Acting Beta 2 Agonist; ICS: Inhaled Corticosteroid.

## Data Availability

The raw data used in this research are available to the authors, depending on INFARMED’s authorization.
